# Multiorgan Metastasis of Human HER-2^+^ Breast Cancer in Rag2^−/−^;Il2rg^−/−^ Mice and Treatment with PI3K Inhibitor

**DOI:** 10.1371/journal.pone.0039626

**Published:** 2012-06-21

**Authors:** Patrizia Nanni, Giordano Nicoletti, Arianna Palladini, Stefania Croci, Annalisa Murgo, Marianna L. Ianzano, Valentina Grosso, Valeria Stivani, Agnese Antognoli, Alessia Lamolinara, Lorena Landuzzi, Emmanuelle di Tomaso, Manuela Iezzi, Carla De Giovanni, Pier-Luigi Lollini

**Affiliations:** 1 Section of Cancer Research, Department of Experimental Pathology, University of Bologna, Bologna, Italy; 2 Rizzoli Orthopedic Institute, Bologna, Italy; 3 Aging Research Centre, “G. D'Annunzio” University, Chieti, Italy; 4 Novartis Institutes for BioMedical Research, Inc., Cambridge, Massachusetts, United States of America; 5 Department of Hematology and Oncological Sciences, University of Bologna, Bologna, Italy; University of South Alabama, United States of America

## Abstract

*In vivo* studies of the metastatic process are severely hampered by the fact that most human tumor cell lines derived from highly metastatic tumors fail to consistently metastasize in immunodeficient mice like nude mice. We describe a model system based on a highly immunodeficient double knockout mouse, Rag2^−/−^;Il2rg^−/−^, which lacks T, B and NK cell activity. In this model human metastatic HER-2^+^ breast cancer cells displayed their full multiorgan metastatic potential, without the need for selections or additional manipulations of the system. Human HER-2^+^ breast cancer cell lines MDA-MB-453 and BT-474 injected into Rag2^−/−^;Il2rg^−/−^ mice faithfully reproduced human cancer dissemination, with multiple metastatic sites that included lungs, bones, brain, liver, ovaries, and others. Multiorgan metastatic spread was obtained both from local tumors, growing orthotopically or subcutaneously, and from cells injected intravenously. The problem of brain recurrencies is acutely felt in HER-2^+^ breast cancer, because monoclonal antibodies against HER-2 penetrate poorly the blood-brain barrier. We studied whether a novel oral small molecule inhibitor of downstream PI3K, selected for its penetration of the blood-brain barrier, could affect multiorgan metastatic spread in Rag2^−/−^; Il2rg^−/−^ mice. NVP-BKM120 effectively controlled metastatic growth in multiple organs, and resulted in a significant proportion of mice free from brain and bone metastases. Human HER-2^+^ human breast cancer cells in Rag2^−/−^;Il2rg^−/−^ mice faithfully reproduced the multiorgan metastatic pattern observed in patients, thus allowing the investigation of metastatic mechanisms and the preclinical study of novel antimetastatic agents.

## Introduction

Metastatic dissemination is the most feared sequel of cancer, and the main cause of mortality. Dissemination from primary tumors can reach every organ in the body, however each tumor type displays a specific metastatic pattern resulting from the interaction of tumor-intrinsic and organ-specific molecular and cellular properties [1], [2]. For example, the most common targets of breast cancer dissemination are the bones, the lungs, abdominal viscera and the brain [3]–[6]. Metastatic patterns determine the duration of recurrence-free intervals and, more importantly, quality of life and overall survival.

The study of metastatic dynamics in cancer patients is constrained by practical and ethical issues, therefore the availability of representative animal models is critical. Unfortunately, the metastatic potential of human tumors and cell lines is expressed incompletely in current animal models [7], [8].

Modifications of experimental conditions brought significant improvements, for example using special injections routes (*e.g.* intracardiac or intracarotid), or cell variants selected for enhanced organ-specific metastasis [9]–[12]. However, such expedients resulted in preclinical models that are further removed from relevant clinical aspects of metastatic dissemination. This problem is acutely felt in the field of metastatic breast cancer, because most studies are based on a handful of metastatic cell lines (in particular MDA-MB-231 and MDA-MB-435), whereas relevant pathological groups, such as HER-2^+^ tumors, lack representative metastatic models. The only solution so far has been to force HER-2 expression in existing HER-2-negative cell lines through gene transduction [13]. However it is unclear whether this approach faithfully models both HER-2 hyperexpression resulting from spontaneous carcinogenesis and the ensuing response of metastatic breast cancer to targeted agents.

An alternative to the manipulation of tumor cells would be the use of more permissive hosts. The major problem with classical nude mice is their intact NK activity, that severely impairs metastatic dissemination [14]. Pioneering studies using different immunodeficient mutants, like *scid* or NIH-III mice, did not reveal sizeable advantages [15], [16]. An initial solution was the pretreatment of nude mice with NK-depleting antibodies [7], however this opened only a narrow temporal window, unsuitable for studies of metastatic dissemination from local tumors, furthermore complete NK depletion could not be reached, especially at the tissue level.

The advent of genetically-modified mice, with specific and stable immune defects, provided novel hosts for the study of human metastatic tumors [3], [8], [17]. We have recently shown that Rag2^−/−^;Il2rg^−/−^ (also called Rag2^−/−^;gamma_c_^−/−^) mice, which lack T, B and NK cells [18], allow the metastatic spread of human musculo-skeletal sarcomas, and are vastly superior to NK-depleted nude mice [7]. We show here that, in Rag2^−/−^;Il2rg^−/−^ mice, the metastatic pattern of human HER-2^+^ breast cancer reproduces the multiorgan dissemination seen in patients, allowing meaningful studies of antimetastatic approaches.

## Results

### Tumor growth and metastatic spread in Rag2^−/−^;Il2rg^−/−^ mice

Many human breast cancer cell lines, in particular those expressing HER-2, grow poorly in nude mice [19], [20], and usually do not metastasize, even when NK activity is temporarily blocked by treatment of the host with NK-depleting antibodies. Table 1 shows that, after subcutaneous injection, HER-2^+^ MDA-MB-453 and BT-474 cells did not give rise to tumors in nude mice (with a minimum follow-up of 15 weeks). The same cells in Rag2^−/−^;Il2rg^−/−^ mice gave rise to progressive local tumors after very short latency times (Table 1), both after subcutaneous and orthotopic (intramammary) administration.

**Table 1:** Tumor growth of human breast cancer cell lines in Rag2^−/−^;Il2rg^−/−^, but not in nude mice.

Mice	Cell line	Route	Tumor incidence	Latency time (wk ± SEM)	Tumor volume^a^ (cm^3^ ± SEM)
Nude	MDA-MB-453	subcutis	0/3^b^	–	–
Rag2^−/−^;Il2rg^−/−^	MDA-MB-453	subcutis	21/21	1.4±0.24	0.90±0.16
	MDA-MB-453	intramammary	8/8	1.8±0.06	0.52±0.16
Rag2^−/−^;Il2rg^−/−^	453-EGFP	subcutis	3/3	1.0±0.00	0.84±0.29
	453-EGFP	intramammary	7/7	1.3±0.18	0.40±0.18
Nude	BT-474	subcutis	0/3^b^	–	–
Rag2^−/−^;Il2rg^−/−^	BT-474	subcutis	10/10	1.7±0.70	2.64±0.35
	BT-474	intramammary	5/5	2.6±1.60	1.72±0.77
Rag2^−/−^;Il2rg^−/−^	474-EGFP	subcutis	6/6	2.7±0.49	2.26±0.68
	474-EGFP	intramammary	6/6	1.0±0.00	1.61±0.59

Abbreviations: wk, weeks; SEM, standard error of the mean.

a Tumor volumes at 12–13 weeks after cell injection, except 474-EGFP at 23 weeks.

b All nude mice were tumor-free 15 weeks after cell injection.

Metastatic dissemination of MDA-MB-453 and BT-474 cells in Rag2^−/−^;Il2rg^−/−^ mice was widespread, and reached all sites commonly affected in human patients, including lungs, liver, bones and brain (Table 2). Multiorgan metastatic dissemination did not show age-related differences within the age range studied. Interestingly, all administration routes led to the development of multiorgan metastases.

**Table 2:** Multiorgan metastatic ability of human breast cancer cell lines in Rag2^−/−^;Il2rg^−/−^ mice.

Cell line	Metastasis origin	Mice with metastases^a^		Mice with multiorgan metastases		Metastatic sites								
						Brain	Lung	Liver	Ovary	Kidney, adrenal	Bone	Bone marrow	Lymph node	Other^b^
MDA-MB-453	Orthotopic tumor	4/4	(100%)	4/4	(100%)	✓	✓		✓	✓		✓		✓
453-EGFP	Orthotopic tumor	11/11	(100%)	10/11	(91%)	✓	✓	✓	✓	✓	✓	✓	✓	✓
BT-474	Orthotopic tumor	2/3	(67%)	1/3	(33%)	✓		✓		✓		✓		
474-EGFP	Orthotopic tumor	6/6	(100%)	4/6	(67%)	✓	✓	✓	✓	✓	✓		✓	✓
MDA-MB-453	Subcutaneous tumor	14/14	(100%)	14/14	(100%)	✓	✓		✓	✓		✓		✓
453-EGFP	Subcutaneous tumor	3/3	(100%)	3/3	(100%)	✓	✓	✓	✓	✓	✓	✓	✓	
BT-474	Subcutaneous tumor	1/8	(13%)	1/8	(13%)	✓	✓		✓					
474-EGFP	Subcutaneous tumor	5/5	(100%)	4/5	(80%)		✓		✓	✓	✓			✓
MDA-MB-453	Intravenous injection^c^	16/16	(100%)	16/16	(100%)	✓	✓	✓	✓	✓		✓		✓
453-EGFP	Intravenous injection^c^	12/12	(100%)	12/12	(100%)	✓	✓	✓	✓	✓	✓	✓	✓	✓
BT-474	Intravenous injection^c^	13/13	(100%)	10/13	(77%)	✓	✓	✓	✓	✓			✓	✓
474-EGFP	Intravenous injection^c^	3/6	(50%)	1/6	(17%)		✓		✓					✓

a Table includes only those mice for which all indicated metastatic sites were homogeneously evaluable, excluding, for example, those cases in which selected organs were separately processed for morphologic or immunohistochemical studies (*see* Fig. 2).

b Other metastatic sites include salivary glands, snout, uterus, interscapular space.

c Mice were sacrificed when they showed signs of distress; mean ± standard error of the mean survival times in weeks were: MDA-MB-453: 9.1±0.32; 453-EGFP: 8.4±0.57; BT-474: 28.9±3.36; 474-EGFP: 59.3±4.92.

**Figure 1:**
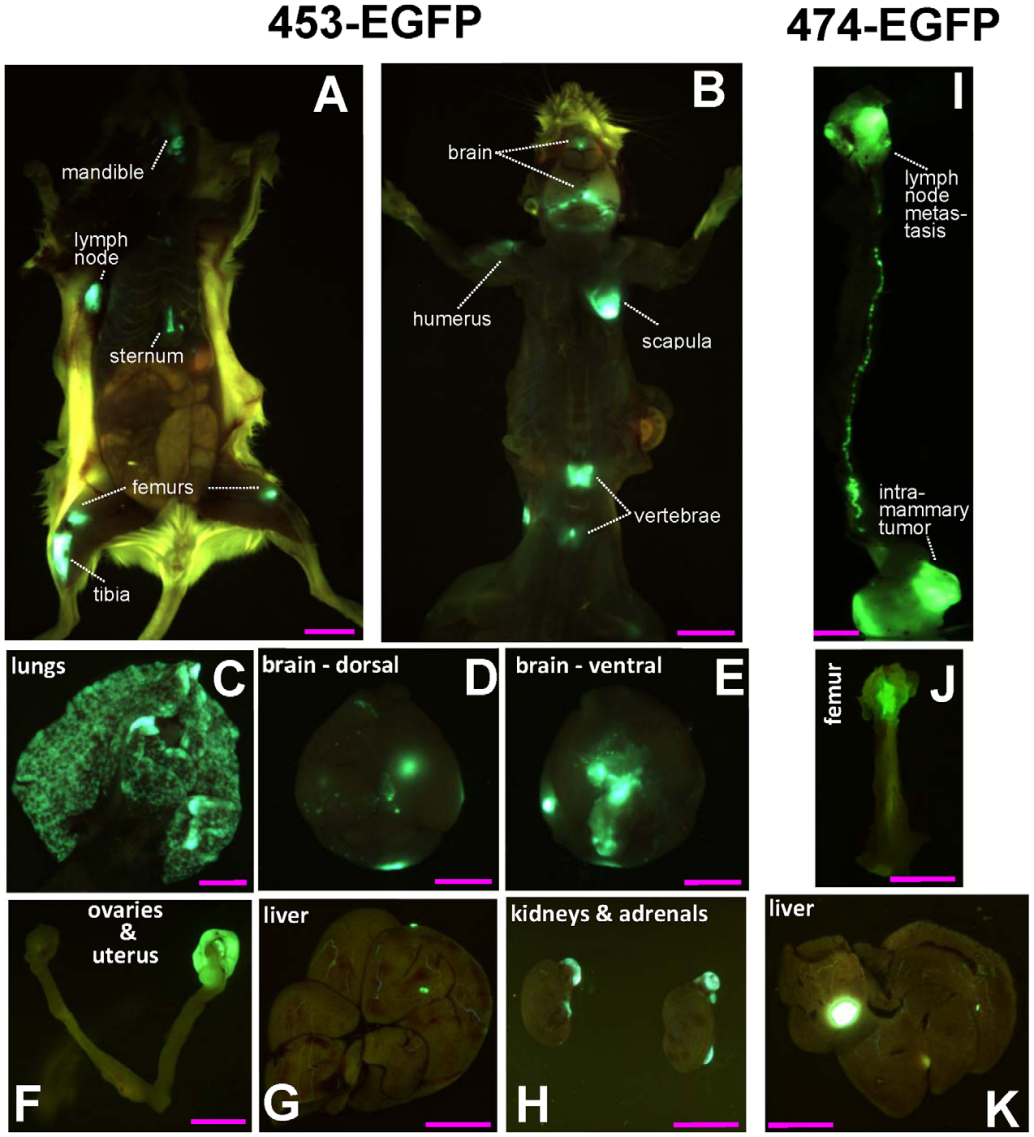
Representative images of metastatic dissemination of human breast cancer cells in Rag2^−/−^;Il2rg^−/−^ mice. In each picture metastases are bright green, clearly distinct from either brownish internal organs or yellow autofluorescence of mouse skin and fur. Panels A-H, 453-EGFP cells; panels I-K, 474-EGFP cells. Routes of injection: intravenous (A, B, G); subcutaneous (D, E, H); intramammary (C, F, I, J, K). (A) Ventral view of a mouse showing multiple metastases prevalently affecting bones. Bar corresponds to 10 mm. (B) Dorsal view of a mouse showing bone and brain metastases. Bar corresponds to 10 mm. (C) Dissected lungs showing widespread metastatic dissemination. Bar corresponds to 3.3 mm. Dorsal (D) and ventral (E) views of a dissected brain with multiple metastases. Bar corresponds to 3.3 mm. (F) Dissected ovaries and uterus with one large ovarian metastasis. Bar corresponds to 5 mm. (G) Dissected liver with metastases. Bar corresponds to 10 mm. (H) Dissected kidneys and adrenal glands with multiple bilateral metastases. Bar corresponds to 10 mm. (I) Dissected primary tumor disseminating from the abdominal mammary gland to an axillary lymph node. Bar corresponds to 5 mm. (J) Dissected femur with metastasis. Bar corresponds to 5 mm. (K) Dissected liver with metastases. Bar corresponds to 9.2 mm.

Different metastatic burdens were observed in different organs, and the use of EGFP-tagged cells was instrumental in allowing a precise detection of metastatic nodules throughout the body. Figure 1 shows representative pictures of multiorgan metastatic dissemination of 453-EGFP (panels A-H) and 474-EGFP (panels I-K) cells in Rag2^−/−^;Il2rg^−/−^ mice. Figures 1A and 1B show two mice with multiple bones metastases, a metastatic localization that became apparent with the use of fluorescence detection. Figures 1C-H show dissected organs with representative pictures of metastatic dissemination to lungs, brain, ovaries, liver, kidneys and adrenals. Figure 1I shows the dissemination of a primary 474-EGFP tumor, growing in an abdominal mammary gland, to an axillary lymph node. Figures 1J-K show a dissected femur and liver with 474-EGFP metastases.

### Brain metastases

The brain is a common site of metastatic spread, but tumor growth in immunodeficient mice fails to reproduce this fateful property of human tumors, unless unique cell lines, selected variants and/or special injection routes are used. In contrast, brain metastases were common in Rag2^−/−^;Il2rg^−/−^ implanted with HER-2+ mammary carcinoma cells, making the Rag2^−/−^;Il2rg^−/−^ mouse an exquisite model for the study of brain dissemination.

**Figure 2:**
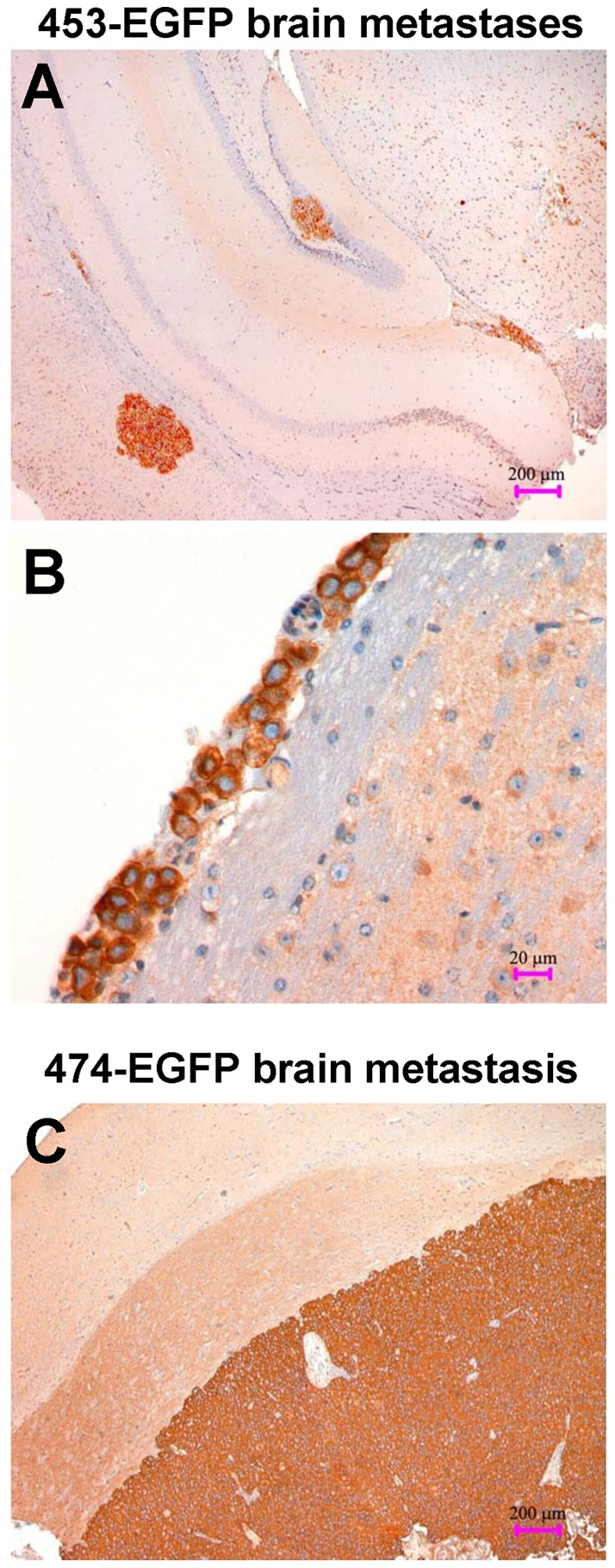
Morphologic aspects of HER-2-positive brain metastases in Rag2^−/−^;Il2rg^−/−^ mice. Immunohistochemical staining for HER-2. (A) Intracerebral metastases after s.c. tumor growth of MDA-MB-453 (magnification x50, bar corresponds to 200 μm). (B) Intraventricular metastasis after s.c. tumor growth of MDA-MB-453 (magnification ×400, bar corresponds to 20 μm). (C) Large cerebral metastasis after i.v. injection of BT-474 cells. (magnification ×50, bar corresponds to 200 μm).

Morphologic analysis of brain deposits stained with anti-HER-2 antibody (Figure 2) showed that mice implanted with MDA-MB-453 (Fig. 2A-B) or BT-474 (Figure 2C) cells had multiple metastases with a distribution recapitulating the clinical situation observed in breast cancer patients developing brain metastasis. Intracerebral and intraventricular or leptomeningeal metastases were detected, ranging from minimal deposits of few cells to prominent masses. Most intracerebral metastases were located in the cerebrum with rare nodules in the cerebellum. As in patients, intracerebral metastases commonly grew at the junction between white and grey matter (cortex and basal ganglia). Leptomeningeal metastases occurred with invasion and proliferation of neoplastic cells in the subarachnoid space. Multifocal infiltration of the leptomeninges in a sheetlike fashion along the surface of the brain and, sometimes, dissemination in the intraventricular spaces were also observed.

### Therapy of disseminated human breast cancer

Metastatic dissemination of human breast cancer is a great therapeutic challenge. Current therapies have led to an improved control of the systemic disease and consequently modified the risk of metastatic relapse in different organs. For example, the monoclonal antibody trastuzumab appears to enhance the risk of brain metastases as first relapse, most likely due to the poor penetration of the blood-brain barrier [21]. New treatments are urgently needed to address this new patient population. A mouse model of multiorgan metastatic dissemination is an important tool to investigate new antimetastatic drugs.

**Figure 3:**
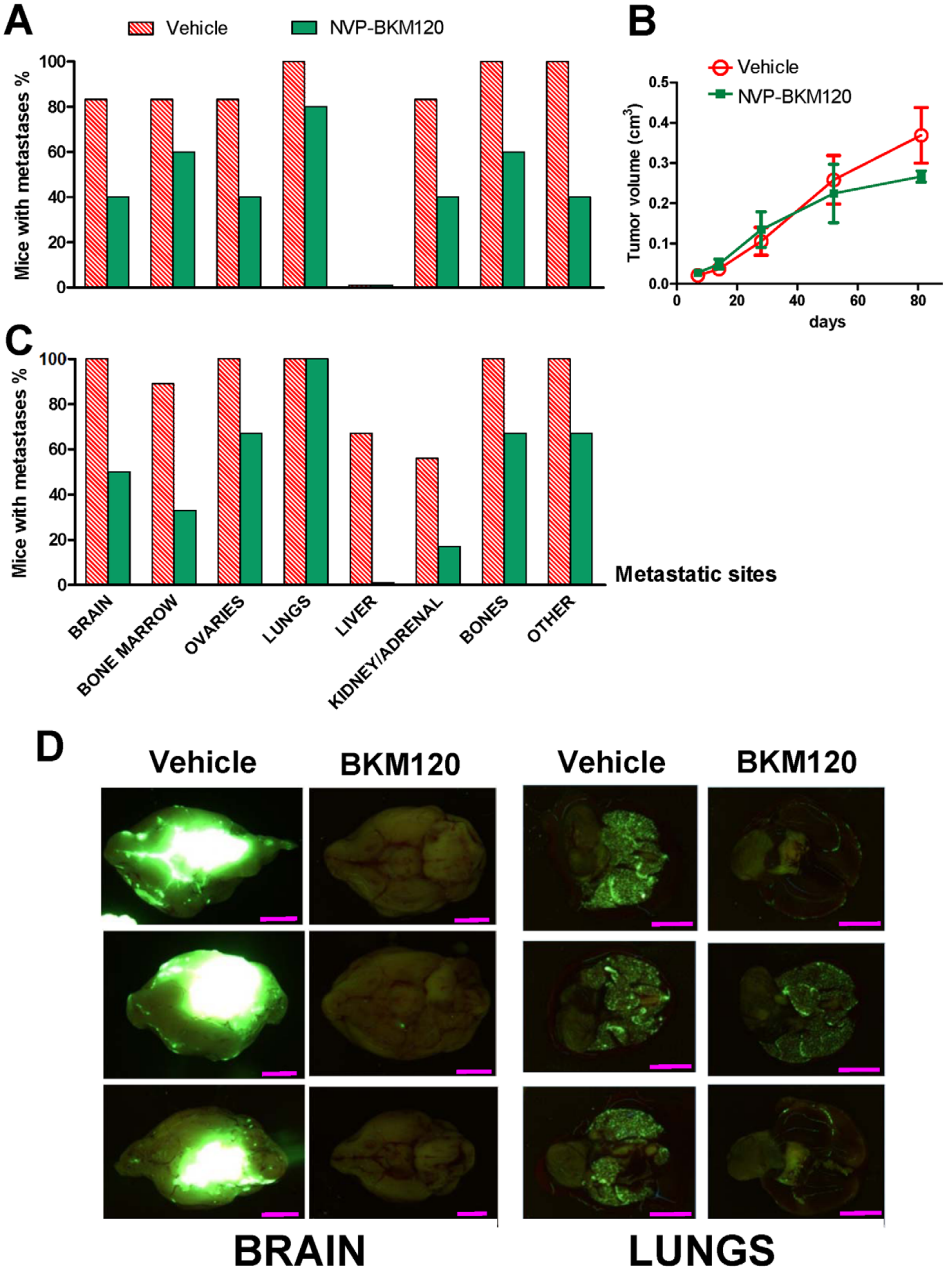
Multiorgan inhibition of 453-EGFP metastatic growth by NVP-BKM120. Incidence of metastases in different sites (A) and local tumor growth (B) after intramammary cell injection. Treatment with NVP-BKM120 started seven days after cell injection. Vehicle, n = 6; NVP-BKM120, n = 5. (C) Incidence of metastases in different sites after intravenous cell injection. Vehicle, n = 9; NVP-BKM120, n = 6. Treatment with NVP-BKM120 started one day after cell injection. A significant inhibition of metastasis by NVP-BKM120 was recorded in the brain, bone marrow and liver, p<0.05 at least, Fisher's exact test. (D) Representative samples from groups of panel C of dissected control and treated mouse brains (ventral view) and lungs showing reduction in metastatic burden by NVP-BKM120. Bars correspond to 2.5 mm (brain) or 7.5 mm (lungs).

We studied whether NVP-BKM120 [22], [23], a selective PI3K inhibitor with a demonstrated penetration of the blood-brain barrier, could control the metastatic growth of 453-EGFP cells in different target organs. Oral NVP-BKM120 produced a strong and widespread reduction in the metastatic burden (Figure 3). The therapeutic activity of NVP-BKM120 was more evident on metastatic localizations (Figure 3A) than on local tumor growth (Figure 3B). In multiple sites a sizeable proportion of mice were noted to be metastasis-free following 7–12 weeks of treatment (Figure 3A and 3C). Even in the more heavily colonized organs, such as the brain, NVP-BKM120 strongly inhibited the growth of 453-EGFP (Figure 3D).

**Figure 4:**
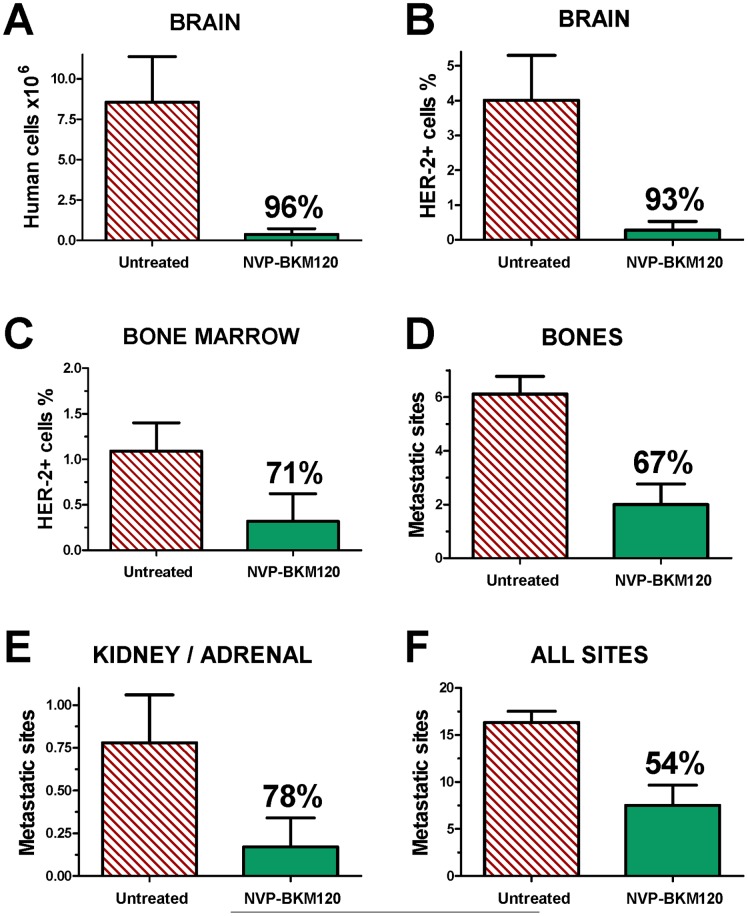
Quantitative analysis of antimetastatic activity of oral NVP-BKM120. Each bar represents the mean and SEM of groups of 6–9 mice treated i.v. with 453-EGFP cells, percentage inhibition is shown in each graph above NVP-BKM120 bar. (A) Metastatic burden in the brain as evaluated by Real-Time PCR, *see* Materials and Methods for calculations; (B, C) Cytofluorometric determination of HER-2^+^ metastatic cells in the dissociated brain (B) and femural bone marrow (C); (D, E, F) Visual count of metastatic sites per mouse, *see* Fig. 1 for representative pictures. Statistical evaluation of metastasis inhibition by NVP-BKM120: panels A, B, D, F, p<0.05 at least by the Student's *t* test.

To better assess NVP-BKM120 therapeutic activity against metastases, we quantitated metastatic cells using human-specific Real-Time PCR or HER-2 flow cytometry. In the brain of mice treated with NVP-BKM120 we found a >90% inhibition in the number of human cells with both technologies (Figure 4A and B), thus confirming the potential of this drug against brain metastases. HER-2+ cells were reduced by 71% in the bone marrow (Figure 4C), and the number of metastatic bone localizations was decreased by 67% (Figure 4D). Metastases to kidneys and adrenal glands were also greatly reduced (Figure 4E). Globally, NVP-BKM120 reduced by more than 50% the number of metastatic sites per mouse (Figure 4F).

## Discussion

We described a model system in which metastatic human cells displayed their full multiorgan metastatic potential, without the need for selections or additional manipulations. In particular, a critical issue in the study of human breast cancer spread, *i.e.* brain metastases, was faithfully reproduced in our system. It is noteworthy that human HER-2^+^ breast cancer cells produced metastases in many organs both after local tumor growth (either orthotopic or subcutaneous) and after intravenous administration.

The results of this work have both practical and theoretical relevance. From a practical point of view, the demonstration that Rag2^−/−^;Il2rg^−/−^ mice are permissive for the metastatic spread of human HER-2^+^ breast cancer solves the long-standing problem of investigating multiorgan metastatic mechanisms of human cancer in preclinical mouse models. Various attempts made in the past to enhance metastatic spread through the use of immunodeficient rodents different from nude mice [15], [24], or of immunosuppressive treatments administered to nude mice [7] had limited success. It is commonplace to study only the few human cell lines that, for unknown reasons, metastasize in nude mice, and to use such cell lines as a platform for the transduction of unexpressed genes, *e.g.* HER-2, and for the selection of more metastatic, or organ-specific cell variants [11], [25]. In summary, previous mouse models of metastatic spread of human tumors were affected by severe shortcomings that could introduce significant biases both in fundamental and in translational studies. Furthermore, in most instances, such models were restricted to a single metastatic localization [26].

We have shown here that Rag2^−/−^;Il2rg^−/−^ mice allow the metastatic spread of human HER-2+ breast cancer cells, recapitulating the clinical situation. We have previously shown that the same is true for an entirely different group of human tumors, musculo-skeletal sarcomas [7]. Altogether our results suggest that Rag2^−/−^;Il2rg^−/−^ mice could unveil the intrinsic tumorigenic and metastatic potential of human cancer cells, which is masked or distorted by the use of poorly permissive hosts. Our results prompt for comprehensive and comparative studies of further tumor histotypes, using both cell lines and primary tumor samples.

An important application of this new *in vivo* model would be the prediction of the metastatic propensity and organ tropism of individual tumors, since current prognostic parameters provide only a probabilistic evaluation. For example, some node-negative breast cancer patients develop metastases, whereas some node-positive patients do not. Furthermore, while the general pattern of metastatic spread is known [1], [4], the specific organ tropism of individual cancers is unpredictable. Such knowledge would be of paramount importance to further the concept of individualized therapies, through organ-focused antimetastatic therapies tailored to individual risk of recurrence. Future studies should investigate whether Rag2^−/−^;Il2rg^−/−^ mice, in combination with molecular signatures and cellular assays, could be used to predict metastatic ability of individual tumors [27].

Relevant conceptual implications of our results concern both metastasis biology and tumor immunity. In the past it was hypothesized that the low metastatic ability of human tumor cells in immunodeficient (nude) mice was caused by inappropriate receptor-counterreceptor interactions involving xenogeneic (*i.e.* human and murine) molecules. If this were the case, to obtain a good metastasis-prone mouse, a myriad of mouse genes would have to be replaced with human equivalents. On the contrary, Rag2^−/−^;Il2rg^−/−^ mice demonstrate that the immune system is the major obstacle to efficient human metastasization in mice, and that a combined immunodeficiency is sufficient to permit the systemic spread of human tumor cells.

When compared to Rag2^−/−^;Il2rg^−/−^ mice, nude mice display an impressive resistance to human tumor cell growth and malignancy (Tab. 1 and [7]), thus suggesting that the differential immunological mechanisms could be exploited for antimetastatic therapies. It is noteworthy that major functional differences between nude and Rag2^−/−^;Il2rg^−/−^ mice, *i.e.* B and NK cells, are underexploited in current immunotherapies [28]. The relevance of B cells and antibodies in cancer immunoprevention and immunotherapy is demonstrated not only by the clinical impact of monoclonal antibodies, but also by a large body of studies focused on HER-2 in mammary carcinoma [29], leading to the development of clinical vaccines that stimulate both T and B cell immunity [30].

The importance of NK cells in the control of metastatic spread has long been recognized [14], however therapeutic applications so far remain at the experimental stage of development. One possible hint for novel developments comes from the fact that systemic treatments against NK cells administered to nude mice had a limited effect on human tumor cell spread, unlike Rag2^−/−^;Il2rg^−/−^ mice which have a generalized NK deficiency. This points to an important role of organ-specific NK cells, a set of NK populations so far not exploited by cancer immunotherapies.

A further advantage of Rag2^−/−^;Il2rg^−/−^ mice is that they can be engrafted with hemopoietic stem cells, thus giving rise to a functioning human immune system [18]. The combination of immune reconstitution and metastatic permissiveness allows for the first time the analysis of natural and adaptive immune responses against human tumors and metastases growing *in vivo*, and the investigation of immunotherapeutic maneuvers aimed at eliciting selected immune activities [De Giovanni et al., manuscript in preparation].

The development of novel antimetastatic drugs is another field to which realistic preclinical models could contribute significant advances [31]. Most drugs currently in clinical use, including small molecules and monoclonal antibodies, underwent *in vivo* preclinical development mainly in nude mice bearing local tumors. Evaluation of organ-specific antimetastatic activity is increasingly important today, as monoclonal antibodies and other targeted therapeutic agents have modified the pattern of metastatic recurrence, and increased the incidence of specific localizations, such as the brain [21], [32], [33].

MDA-MB-453 breast cancer cells used in this work were resistant to the antiproliferative activity of trastuzumab *in vitro* (our unpublished results and [34]), therefore the results shown here modeled a relevant clinical situation in which HER-2^+^ breast cancer progressed in the brain and other organs after anti-HER-2 monoclonal antibody therapy. The efficacy of NVP-BKM120 in controlling metastatic growth in multiple organs, including the brain, forecasts clinical impact in analogous clinical situations of this novel oral pan-PI3K inhibitor.

## Materials and Methods

### Ethics Statement

All experiments were performed according to Italian and European guidelines and were specifically authorized by the Animal Care and Use Committee of the University of Bologna (project sent to the Italian Ministry of Health with letter n. 12511-X/10, supervisor: Prof. Carla De Giovanni).

### Mice

Rag2^−/−^;Il2rg^−/−^ (elsewhere referred to as Rag2^−/−^;gamma_c_^−/−^) breeders were kindly given by Drs. T. Nomura and M. Ito of the Central Institute for Experimental Animals (Kawasaki, Japan). Nine–20-week-old female Rag2^−/−^;Il2rg^−/−^ mice, bred in our animal facilities under sterile conditions, were used throughout this work. Six-8-week-old female athymic Crl: CD-1-*Foxn1* ^nu/nu^ mice (referred to as nude mice) were purchased from Charles River Italy and kept under sterile conditions.

### Cell lines

MDA-MB-453 [35] and BT-474 [36] breast cancer cell lines were originally obtained from Dr. Serenella M. Pupa (Istituto Nazionale dei Tumori, Milan, Italy). Cell lines were authenticated by DNA fingerprinting on the 11^th^ November 2010 (performed by DSMZ, Braunschweig, Germany). Cells were routinely cultured in Roswell Park Memorial Institute (RPMI) medium supplemented with 10% foetal bovine serum and were maintained at 37°C in a humidified 5% CO_2_ atmosphere. All medium constituents were purchased from Invitrogen, Milan, Italy. To visualize metastatic deposits, breast cancer cell lines were transfected with a plasmid expressing Enhanced Green Fluorescent Protein (pEGFP-N1, Clontech, Mountain View, CA) using Lipofectamine 2000 (Invitrogen). Stable transfectants were selected using G418 (Invitrogen). EGFP expression was monitored by fluorescence microscopy and quantified by cytofluorometric analysis.

### Metastasis induction

Local tumors were induced with 10^7^ viable human cells injected either subcutaneously in the right hind leg in 0.2 ml of PBS, or in the fat pad of the fourth (abdominal) left mammary gland in 0.1 ml of PBS. Intramammary administration was performed by reaching with a 29-gauge syringe needle the area underlaying the nipple and the areola, and observing the formation of a swelling under that area (modified from [37]). Tumor diameters were periodically measured with digital calipers; tumor volumes were calculated as π/2·[√(*a·b*)]^3^/6, where *a* = maximal tumor diameter and *b* = major tumor diameter perpendicular to *a*. For intravenous (i.v.) administrations, 2 million cells in 0.4 ml PBS were injected in a tail vein. Pilot experiments were performed to assess for each cell line the time at which experimental metastases could be detected.

### Metastasis detection and quantification

Tumor-bearing mice were sacrificed at various times (*see* Results), depending on tumor and metastasis growth, and were subjected to an accurate necropsy. Lungs were perfused with black India ink to better outline metastases and fixed in Fekete's solution. Lung and liver metastases were counted using a dissection microscope. When EGFP-expressing cells were injected, whole mice and dissected organs were carefully examined using a Lightools imaging system (Lightools Research, Encinitas, CA) to detect fluorescent metastatic deposits. Quantification of metastatic load in brain and bone marrow was performed by immunofluorescence followed by cytofluorometric analysis and by Real-Time PCR. Brain was minced with scissors and passed through a 70 μm cell strainer (Becton Dickinson, Bedford, MA, USA) to obtain a homogeneous cell suspension. Bone marrow was flushed from both femurs in PBS and filtered through a 70 μm strainer. A mouse monoclonal antibody against human HER-2 (clone Neu 24.7, Becton Dickinson, San Jose, CA, USA) labeled with phycoerythrin was used to quantify human cells by cytofluorometric analysis. Real-Time PCR was performed on genomic DNA extracted with 10 mM Tris-HCl buffer pH 8.3 containing 50 mM KCl, 2.5 mM MgCl_2_, 0.01% gelatin, 0.45% igepal, 0.45% tween 20 and 120 μg/ml proteinase K (all reagents from Sigma, Milan, Italy) by an overnight incubation at 56°C followed by 30 min incubation at 95°C to inactivate the proteinase K. A sequence of the α-satellite region of the human chromosome 17 was amplified. Primer and probe sequences were derived from Becker et al. [38], with the sole alteration that the probe carried the non-fluorescent quencher dye TAMRA at the 3′-end. A 100 ng DNA aliquot per sample was amplified using 250 nM primers and 100 nM probe in a final volume of 25 μl of TaqMan Universal PCR Master Mix (Applied Biosystems, Milan, Italy). After an initial denaturation step at 95°C for 10 min, 45 cycles of amplification (95°C for 30 sec plus 60°C for 1 min) were performed in a 5700 Sequence Detection System (Applied Biosystems). DNA extracted from mouse tissues showed no amplification up to 45 cycles. To quantify human cells, a standard curve was constructed by adding scalar amounts of MDA-MB-453 human cells to cells from the mouse whole brain. C_t_ (threshold cycle) values obtained from the experimental samples were interpolated in the standard curve run in each PCR.

### Anti-metastasis therapy with NVP-BKM120

NVP-BKM120 (Novartis Institutes for BioMedical Research – Oncology, Basel, Switzerland) was formulated in 1-methyl–2-pyrrolidone (NMP)/poly–ethylene glycol 300 (Fluka) (10/90, v/v). Solutions (7.5 mg/ml) were prepared fresh each day of treatment and carefully shielded from light. Groups of 5–9 Rag2^−/−^;Il2rg^−/−^ female were challenged with 453-EGFP cell either intramammary or i.v. A dose of 50 mg/kg NVP-BKM120 was given by gavage daily starting one or seven days after cell injection, control mice received vehicle alone. Mice received four drug administrations in the first week and five drug administrations in the following weeks, for a total of 12 weeks (intramammary tumor cell challenge) or 7 weeks (i.v. challenge).

### Immunohistochemistry

Sections of formalin-fixed, paraffin-embedded samples (2.5 μm thick) were heated to 42°C and dehydrated in xylene and graded alcohols. Antigen retrieval was performed in 10 mM citric acid pH 6 for 6 minutes at 125°C in a Decloaking Chamber (Biocare Medical, Concord, CA). Slides were allowed to cool for 5 minutes in ice, followed by repeated rinsing with distilled water. Slides were incubated with a 1∶800 dilution of a rabbit polyclonal anti-Human c-erbB-2 Oncoprotein (Dako Corporation, Carpinteria, CA – USA), followed by a biotinylated goat anti – rabbit IgG (Jackson ImmunoResearch Laboratories, Inc., West Grove, PA) secondary antibody at 1∶500 dilution. Immunoreactive antigens were detected using Streptavidin Peroxidase (Thermo Scientific – Lab Vision Corporation, Fremont, CA – USA) and DAB Chromogen System (Dako).
